# Effect of Borax On Sintering Kinetics, Microstructure and Mechanical Properties of Porous Glass-Ceramics From Coal Fly Ash by Direct Overfiring

**DOI:** 10.3389/fchem.2022.839680

**Published:** 2022-03-17

**Authors:** Li Zeng, Hongjuan Sun, Tongjiang Peng

**Affiliations:** ^1^ School of Architecture and Civil Engineering, Chengdu University, Chengdu, China; ^2^ Key Laboratory of Solid Waste Treatment and Resource Recycle, Ministry of Education, Southwest University of Science and Technology, Mianyang, China

**Keywords:** coal fly ash, borax, overfiring, porous glass-ceramics, viscous flow

## Abstract

The direct sintering process of coal fly ash for the preparation of glass-ceramics is the liquid-phase sintering process, from non-densification to densification. When the temperature exceeds the densification temperature point, the porosity of glass-ceramics on the contrary increases and the pore diameter increases. This provides a basis to prepare porous glass-ceramics by direct overfiring. Adding borax to coal fly ash can reduce the temperature of liquid phase formation, reduce the preparing temperature of porous glass-ceramics, achieve the purpose of energy saving. The effects of borax on the structure, properties and sintering kinetics of porous glass-ceramics prepared from coal fly ash by overfiring were investigated. It is found that the introduction of B-O bond can change the network structure of non-crystalline vitreous in coal fly ash, reduce the melting temperature, promote the formation of liquid phase, and thus increase the porosity of porous glass-ceramics. This paper provides a certain experimental basis for the preparation of porous glass-ceramics by direct overfiring of coal fly ash at low temperature without adding pore-forming agent, and provides a new possibility for the high-value resource utilization of coal fly ash.

## Introduction

Porous glass-ceramics is a new kind of material which distributes a large number of microcrystalline phases and pores evenly in glass phase based on glass-ceramics. Porous glass-ceramics has been widely used as insulation materials for energy-saving building walls and thermal pipelines due to its large specific surface area, better thermal stability, lower thermal conductivity and higher mechanical strength (Flesoura et al., 2021; Yio et al., 2021). At present, the preparation methods of porous glass-ceramics mainly include crystallization-acid leaching (Wang et al., 2003a; Wang et al., 2003b; Chen et al., 2020), melting sintering with pore-making agent (Ding, et al., 2015; Jiang et al., 2017), direct sintering with pore-making agent (Bernardo and Albertini, 2006; Chen et al., 2016; Hisham et al., 2020; Yio et al., 2021). Among them, the preparation methods of porous glass-ceramic with coal fly ash as raw material are mainly the melting sintering method with adding pore-making agent and the direct sintering method with adding pore-making agent.

The melting sintering method with adding pore-making agent is to add pore-making agent to the basic glass. In the sintering process, pore-making agent through oxidation or release gas to form pores to prepare porous glass-ceramics. The preparation process is similar to that of preparing glass-ceramics by melting and sintering, which includes batching, melting, water quenching, drying, crushing, sieving, forming, nucleation, crystallization and pore forming (Zhu et al., 2012). As environmental protection is deeply rooted in people’s mind, people put forward new requirements for resource utilization (Zeng et al., 2021). In order to save energy consumption and simplify the preparation process, the porous glass-ceramics was prepared by adding pore-making agent in the direct sintering process (Bai et al., 2014; Li et al., 2018; Zhu et al., 2016). No matter which method is used to prepare porous glass ceramics, the type and quantity of pore-forming agent are important indicators affecting the porous glass ceramics (Fernandes et al., 2009; Hou et al., 2017; Wang et al., 2018; Fernandes et al., 2020). The key of pore-making agent selection is that the pore-making temperature should be consistent with the softening temperature of the raw material (Wu et al., 2006). However, the composition and properties of coal fly ash from different producing areas vary greatly. Therefore, it is difficult to select the appropriate type and amount of foaming agent according to its softening temperature point.

Dong et al. found that coal fly ash would self-expansion in the high-temperature sintering process, that is, crystals would be precipitated from the molten liquid to form pores in the high-temperature process (Dong et al., 2008; Dong et al., 2009). Moreover, in the previous experiments, it was found that, the process of sintering is actually the transformation of open pore porosity between particles and particles in the sample into closed pore porosity. In this process, there is an optimal temperature point for densification. Once the temperature point is exceeded, the samples will be overfiring and more molten liquid will be formed. With sufficient viscous flow, small pores will be merged to form large pores, and more crystals will be precipitated from the molten liquid, and the total porosity will gradually increase (Zeng et al., 2020; Zheng et al., 2020). This provides a basis for direct overfiring of porous glass-ceramics. However, preliminary experimental results show that the overfiring temperature of coal fly ash reaches about 1,190°C (Zeng et al., 2019). In order to reduce the temperature of porous glass ceramics prepared by overfiring, a fluxing agent must be added. Borax is widely used as a flux in the preparation of porous glass ceramics, it can reduce the temperature of liquid phase formation (Chen et al., 2013; Guo et al., 2014; Yio et al., 2021). In this experiment, borax was added to reduce the temperature of preparing porous glass-ceramics prepared by overfiring method, so as to achieve the purpose of energy saving and consumption reduction.

The purpose of this paper is to discuss the effect of borax on the morphology, performance and sintering kinetics of the coal fly ash porous glass-ceramics prepared by overfiring, so as to achieve the purpose of preparing porous glass-ceramics without adding pore-making agent at low temperature, and to provide a new way for the high-value utilization of coal fly ash.

## Experimental Procedure

### Raw Materials and Experimental Equipment

**FIGURE 1:**
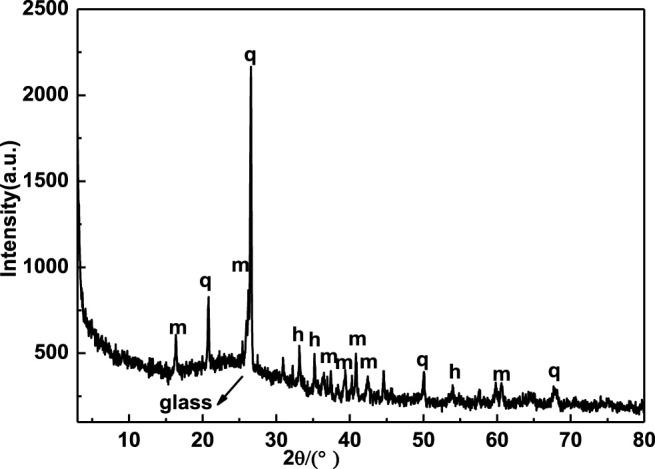
XRD spectra of coal fly ash (q, quartz; m, mullite; h, hematite).

Raw materials, the coal fly ash used in the experiment came from a coal-fired power plant in Jiangyou, Sichuan, the pure polyvinyl alcohol (PVA) and borax were purchased from Chengdu Kelon Chemical Co., LTD. The chemical composition analysis and phase composition of coal fly ash are shown in Table 1 and Figure 1 respectively. The main constituents of coal fly ash are alumina and silicon oxide, accompanied by a small amount of calcium oxide and iron oxide. The phase composition is mainly the amorphous silicate vitreous represented by the uplift steamed bread peak, crystalline mullite (PDF#00-002-0430,3Al_2_O_3_.2SiO_2_), quartz (PDF#00-033-1161, SiO_2_) and hematite (PDF#00-024-0072,Fe_2_O_3_) (Lin et al., 2015).

**TABLE 1:** Chemical compositions of coal fly ash powder.

Composition	SiO_2_	Al_2_O_3_	CaO	Fe_2_O_3_	K_2_O	SO_3_	TiO_2_	Na_2_O
wt%	53.31	26.55	5.88	4.41	2.42	1.42	1.06	0.60
Composition	MgO	P_2_O_5_	BaO	MnO	ZrO_2_	ZnO	Other	LOI
wt%	0.52	0.47	0.10	0.03	0.03	0.02	0.16	3.02

Experimental equipment, 769YP-24B infrared powder tablet press (Xi ‘an minsks testing equipment Co. Ltd., China), KSY-25-16-8X2-20-13 box type resistance furnace (Shanghai shiyan electric furnace, Co. Ltd., China), 305F-2 microcomputer control electronic pressure testing machine (Shenzhen wance testing machine Co. Ltd., China), WT5003GHS hydrostatic balance (Changzhou Wantai Balance Instrument Co. LTD.), XPM-Ф100 × 4 planetary four-cylinder grinding machine (Wuhan prospecting machinery Factory).

### Preparation of Porous Glass-Ceramics

The specific process of preparing porous glass-ceramics by direct overfiring with borax is shown below. Borax (Na_2_B_4_O_7_ 10H_2_O) and coal fly ash were mixed evenly according to mass ratios of 10:90, 15:85, 20:80, 25:75 and 30:70 (denoted as 10B-90F, 15B-85F, 20B-80F, 25B-75F, 30B-70F) for 5 min by ball grinding. PVA was used as the binder for granulation. The samples were sintered in a box type resistance furnace at different temperatures (800–1100°C) for 60 min after tablet forming. After cooling down, the samples were taken out for phase, morphology and corresponding performance test and characterization. The sample was labeled as 10B-90F, 15B-85F, 20B-80F, 25B-75F, 30B-70F sintering temperature.

### Characterization

The phase composition of the raw material and the samples were analyzed by the X-ray diffractometer (XRD) of PANalytical B.V. (2θ range 3–80°, step 0.03°). The chemical composition of raw materials was analyzed by X-ray fluorescence (XRF) of PANalytical B.V. (Rh target, maximum power 2.4 kW). The morphology of porous glass-ceramics was characterized by DV230E3FL optical microscope and the pore size distribution was measured by “Nano Measurer” size statistics software. The three-point bending strength of 5 × 10 × 60 mm^3^ samples was tested at the speed of 0.05 mm min^−1^, and the average value was taken three times for each test. The true density ( ${\text{ρ}}_{\text{t}}$ ) and bulk density ( ${\text{ρ}}_{\text{b}}$ ) were measured by the gas (A_r_) pycnometer and Archimedes method, respectively. The porosity (P) can be calculated by Eq. 1 (Bai et al., 2014).

$$\text{P}=\left(1-\frac{{\text{ρ}}_{\text{b}}}{{\text{ρ}}_{\text{t}}}\right)\times 100\text{\%}$$ (1)

## Results and Discussion

### Phase and Microstructural Evolution

**FIGURE 2:**
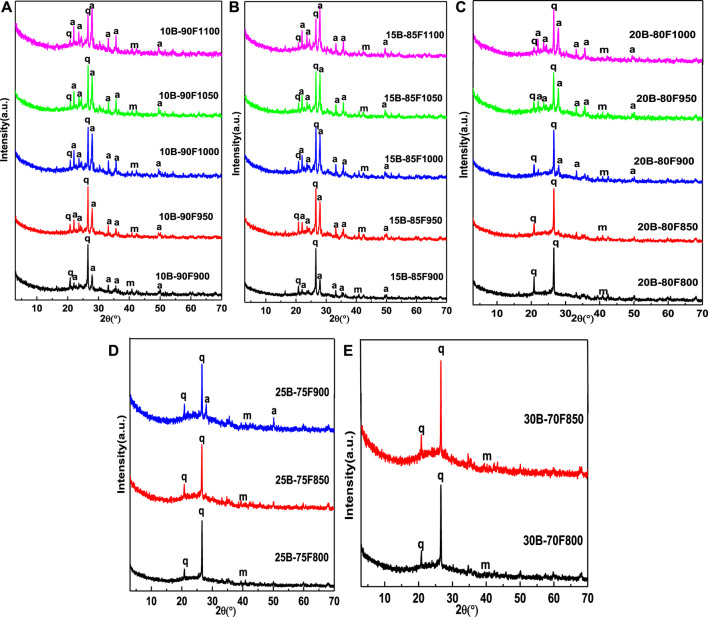
XRD patterns of sintered samples with different borax additions at different temperature (q-quartz, m-mullite, a-anorthite).

Figure 2 shows the XRD patterns of samples sintered at different temperatures with different borax additions. It can be seen from the figure that the addition amount of borax has a great influence on the phase of porous glass-ceramics. When the amount of borax is small (10B-90F, 15B-85F), the phase of the porous glass-ceramics are the same as the glass-ceramics prepared without borax, they all composed of anorthite, quartz and mullite (Zeng et al., 2019). Without borax, anorthite is precipitated from molten quartz and amorphous vitreous at a temperature of 1,000°C (Zeng et al., 2019). When the amount of borax is 10% and 15%, the temperature of anorthite precipitated reduced to 900°C. Indicating that borax will react with the oxides in coal fly ash to reduce the melting temperature of quartz and amorphous vitreous in coal fly ash, so that the molten liquid phase is formed at a lower temperature, and anorthite precipitated (Chen et al., 2013). By comparing the peak strength ratio of anorthite (I_204_) and quartz (I_101_) between 10B-90F1000 and 15B-85F1000 at 1,000°C, it is found that the ratio increases from 0.87 of 10B-90F1000 to 0.91 of 15B-85F1000. Meaning that borax can reduce the melting temperature of quartz and amorphous vitreous, and increase the amount of anorthite precipitated from the molten liquid. By comparing the critical temperature point of collapse of porous structure of porous glass-ceramics (10B-90F is 1,100°C, 15B-85F is 1,100°C, 20B-80F is 1,000°C, 25B-75F is 900°C), it is found that with the increase of borax addition, the critical temperature point of collapse of porous structure decreases gradually. In addition, by comparing the peak strength ratio of anorthite (I_204_) and quartz (I_101_) at the critical temperature point, it was found that the ratio increased from 1.12 in 10B-90F1100 to 1.32 in 15B-85F1100, and then decreased to 0.68 in 20B-80F1000 and 0.47 in 25B-75F900. The reason may be that, at the low amount of borax (10B-90F, 15B-85F), due to the higher critical temperature point of porous structure collapse (1,100, 1,100°C), the addition of borax can simultaneously reduce the melting temperature point of quartz and amorphous vitreous in coal fly ash to form liquid phase, precipitation of more anorthite. With the addition of borax (20B-80F, 25B-75F), the collapse temperature of porous structure reduced (1,000, 900°C). At this temperature, the addition of borax can only reduce the melting temperature of non-crystalline vitreous body in coal fly ash, cannot promote the melting of quartz in coal fly ash. Therefore, the content of precipitated anorthite is lower than that of 10B-90F and 15B-85F. Continue to increase the amount of borax (30B-70F), no anorthite was observed before the pore structure of porous glass-ceramics collapsed (850°C), meaning the glass liquid phase is formed by the melting of borax. This is also the reason why borax is not detected in XRD (Chen et al., 2012). Under the proper temperature, the B-O bond with low binding energy in borax can changes the network structure of quartz and amorphous vitreous in coal fly ash to form liquid phase (Chen et al., 2012). Therefore, the sintering temperature also plays an important role in the liquid phase formation of porous glass-ceramics.

**FIGURE 3:**
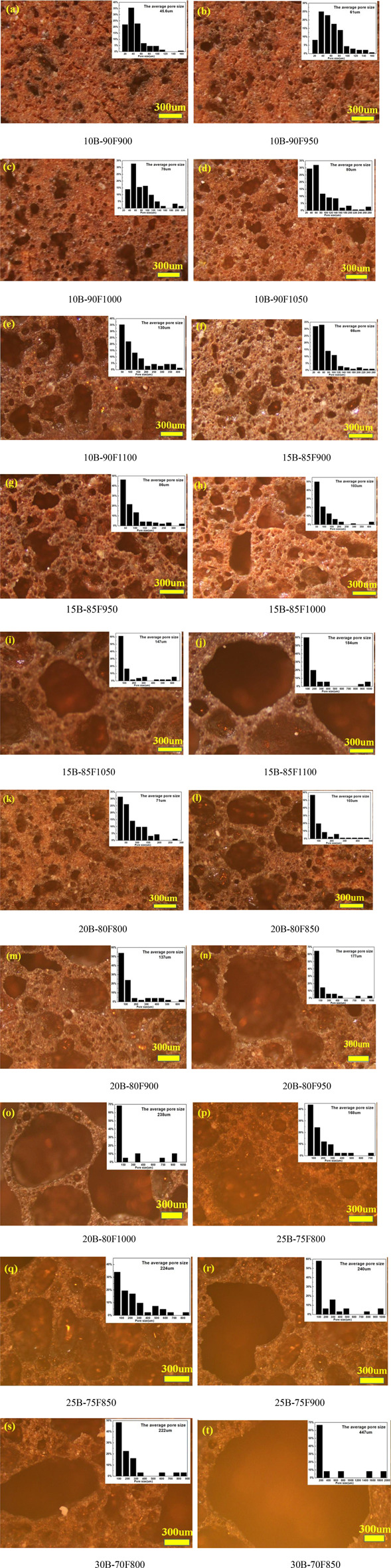
Optical microscopy of sintered samples with different borax additives at different temperatures.

Figure 3 shows the influence of sintering temperature on the morphology and pore size distribution of porous glass-ceramics under different borax additions. Figures 3A–E shows the change of the morphology of the sample with temperature when borax is added at 10%. The average pore size increased from 46 μm in 10B-90F900 to 130 μm in 10B-90F1100, and the distribution of pore size increased from 20–160 μm in 10B-90F900 to 20–430 μm in 10B-90F1100. Figures 3F–J is the morphology and pore diameter distribution of the samples at 900–1,100°C when the addition amount of borax is 15%. By comparing Figures 3A–J, the average pore diameter and pore diameter range of the samples increase with the addition of borax at the same temperature. For example, the average aperture of 10B-90F1000 is 78 μm, the aperture range is 20–220 μm, the average aperture of 15B-85F1000 increases to 103 μm, and the aperture range is 20–400 μm. On one hand, the increase of borax provides more molten liquid, which reduces the viscosity, promotes the formation of pores and the merging of small pores. On the other hand, the introduction of borax destroyed the quartz and amorphous glass structure of coal fly ash (Zeng et al., 2019), reduced its melting temperature, and formed more liquid phase to promote the formation of pores. The anorthite precipitated from the molten liquid phase. Therefore, the results are consistent with Figures 2A,B, at the same temperature, the amount of anorthite in the sample with the borax additive amount of 15% was higher than that of 10%.

The micromorphology and pore size distribution of the porous samples were shown in Figures 3K–R, when the addition amount of borax was 20% and 25%, respectively. It can be seen from the figure that no matter how much borax is added, the average diameter and diameter distribution of the samples increase with the increase of temperature. For example, the average particle size of 20B-80F800 is 71 μm, and the particle size distribution range is 20–280 μm. When the temperature increases to 900°C, the average particle size of 20B-80F900 is 137 μm, and the particle size distribution range is 20–600 μm. As another example, the average particle size of 25B-80F800 is 168 μm, the particle size distribution range is 20-700 μm. The average particle size of 25B-80F850 is 224 μm and the particle size distribution range is 20–800 μm when the temperature increases to 850°C. Interestingly, the average aperture of the 20B-80F1000 is 238 μm and the aperture range is 50–1,000 μm, while the average aperture of the 25B-75F900 is 240 μm and the aperture range is 50–1000 μm. It means that the two have the same liquid phase and the same viscosity at different temperatures and different borax addition. By comparing Figures 2C,D, the content of anorthite in 20B-80F1000 is higher than in 25B-75F900, indicating that more liquid phase is provided by coal fly ash in 20B-80F1000, so more anorthite is precipitated out. More liquid phase is provided by borax in 25B-75F900, means increase the amount of borax can decrease the sintering temperature of porous glass-ceramics. So, to obtain porous samples with the same pore size and pore size distribution, borax can be added or sintering temperature can be increased. Figures 3S,T show the micromorphology and pore diameter distribution of 30B-70F800 and 30B-70F850. In combination with Figure 2E, no anorthite was observed in the sample when the borax addition amount was 30%. Due to the addition amount of borax is large, borax has been completely melted and form a liquid phase to promote the formation of pores before destroy the quartz and amorphous glass structure in coal fly ash. The average pore size of the sample at 850°C has reached 447 μm. In conclusion, the addition of borax can increase the high temperature liquid phase, reduce the viscosity and promote the formation of pores. At the same time, it can increase the average pore size and pore size distribution range of porous glass-ceramics, reduce the sintering temperature of porous glass-ceramics, reduce energy consumption.

### Properties

**FIGURE 4:**
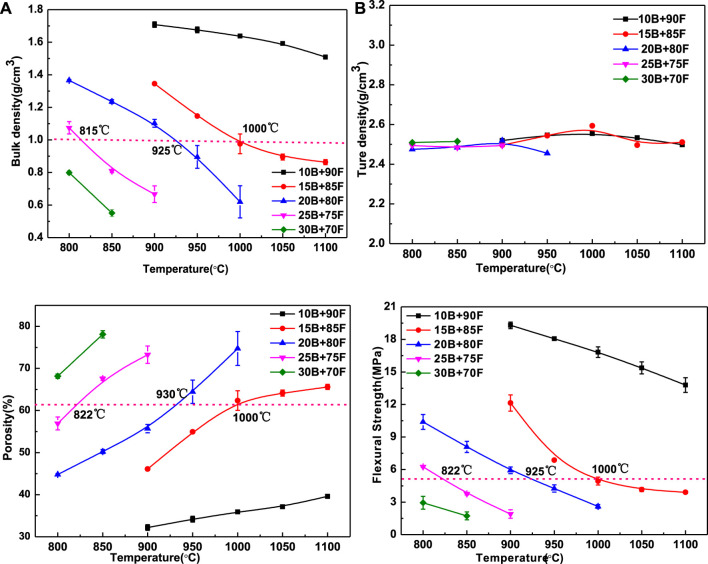
The bulk density **(A)** true density **(B)**, porosity **(C)** and flexural strength **(D)** of porous glass-ceramics with different borax additions vary with temperature.

Figure 4 shows the effect of borax addition amount on the performance of porous glass-ceramics, in which 4(1) is bulk density, 2) is true density, 3) is porosity, and 4) is flexural strength. Obviously, the amount of borax has great influence on the bulk density, porosity and flexural strength of porous glass-ceramics, but has little effect on its true density. The main reason is that the true density is not affected by the porosity, but related to the phase. As can be seen from Figure 2, although the phases contained in porous glass-ceramics are different, the difference in true density between phases is small, so the addition of borax amount has little impact on the true density of porous glass-ceramics (Figure 4B). The bulk density and porosity of porous glass-ceramics are closely related to the viscosity of vitreous. With the increase of borax addition and temperature, the viscosity of vitreous decreases gradually, porosity increases gradually, and bulk density decreases gradually (Figures 4A,C). The similar average particle size and the same particle size distribution of 20B-80F1000 and 25B-75F900 in Figures 3O–R, meaning that the viscosity of the two high-temperature liquid phases is basically the same. It can be seen that the addition amount of borax and sintering temperature have a great influence on the viscosity of high temperature liquid phase in the preparation process of porous glass-ceramics. Similarly, using the bulk density of 1.0 g/cm^3^ as the ordinate plot in Figure 4A, showing that in order to get the bulk density of 1.0 g/cm^3^ porous glass-ceramics, relatively low sintering temperature of 815°C but high borax content 25% can be used, or a moderate sintering temperature of 925°C and moderate borax content 20% can be selected, or a relatively high temperature of 1,000°C and relative lower borax content 15% can be used. The same trend can be seen in Figure 4C. The porosity of porous glass-ceramics sintered at 1,000°C and with borax addition of 15% is the same as that sintered at 925°C and with borax addition of 20%, sintered at 815°C and with borax addition of 25%. It means that the sintering temperature and the amount of borax can adjust the viscosity of the high temperature liquid phase, which has a great influence on the bulk density and porosity of porous glass-ceramics.

The flexural strength of porous glass-ceramics depends on its bulk density and porosity. The larger the bulk density is, the greater the flexural strength is, and the greater the porosity is, the smaller the flexural strength is (Bernardo et al., 2010). It can be seen from Figure 4D that, on the whole, with the increase of temperature and borax addition, the flexural strength decreases. As can be seen from the dotted line in Figure 4D, when the sintering temperature is 822, 925 and 1,000°C, the amount of borax added is 25%, 20% and 15%, the porous glass-ceramics has the same flexural strength. It means that the flexural strength of porous glass-ceramics is actually affected by the viscosity of high-temperature liquid. Therefore, in the practical application process, the viscosity of high temperature liquid can be adjusted by adjusting the sintering temperature or borax addition, so as to adjust the bulk density, porosity and flexural strength of porous glass-ceramics.

Table 2 compares the properties of porous materials prepared from coal fly ash. After comparison, the amount of coal fly ash used in this experiment is relatively high, ranging from 70% to 90%, with a wide range of porosity, bulk density and flexural strength. The porous materials prepared in this experiment can be adjusted according to their properties of porosity, bulk density and flexural strength. More importantly, the porous materials prepared in this experiment without the pore forming agent, and there was no problem in choosing the amount of the type of pore forming agent.

**TABLE 2:** Comparison of properties of porous materials prepared from coal fly ash.

Samples	Raw material	Porosity (%)	Flexural strength (MPa)	Bulk density (g/cm^3^)	Compressive strength (MPa)	References
Foam glass ceramic	30% HAFA, 50% Waste glass, 15% clay and 5% Feldspar (2% CaSO_4_)	—	—	0.98	9.84	Wang et al., 2018
Porous glass- ceramics	55% solid (fly ash:glass-1:1), 27% water glass, 18% dolapix CE 64 (Using polyurethane foam as pore creators)	70	4.5	—	—	Bossert et al., 2004
Foam glass	14.75% fly ash and 84.75% waste glass (0.5% SiC)	81.55	-	0.2672	0.9829	Bai et al. (2014)
Foam glass	50-70% fly ash, 5% Na_2_HPO_4_, 22.5-37.5% Na_2_B_4_O_7_ and 7.5-12.5% CaCO_3_	52-66.1	1.95-2.59	0.591-0.876	2.09-3.95	Chen et al
Foam glass	20% fly ash and 80% glass (1-5% carbonates)	—	—	0.36-0.41	2.40-2.80	Femandes et al., 2009
Foam ceramics	26.25–40% fly ash, 40–50% red mud, 15–20% sodium borate and 5% sodium silicate	64.14-74.15	2.31-8.52	0.51-0.64	4.04-10.63	Chen et al., 2013
Porous glass- ceramics	70-90% coal fly ash and 10-30% borax	32.2-78.1	1.72-19.29	0.55-1.71	—	This paper

### Sintering Kinetics

According to Kingery’s liquid-phase sintering theory, liquid-phase sintering can be divided into three stages: with the increase of sintering temperature, a large number of liquid phase appeared, and the solid particles precipitated from the liquid phase moved and rearranged under the action of capillary force; further rearrangement of grain creep; through liquid phase mass transfer, the grain size and grain shape change constantly, and the rearrangement achieves densification (Luo et al., 2019). In previous experiment, it was found that rise temperature after the sample was densified, the small pores originally existing in the sintered sample would be merged into large pores to form porous structure due to the effect of mass transfer in liquid phase (Zeng et al., 2020). The formation temperature of porous glass-ceramics is between overfiring and softening of the sample, so the sintering activation energy cannot be calculated by the shrinkage rate of the sample with temperature. With the progress of sintering, the porosity of the sample increases and the bulk density decreases. The dynamic empirical formula of ceramic sintering can be used to calculate the activation energy of porous glass-ceramics (Eq 2, 3).

D=Klogt+C (2)

$$\text{K}=\text{Aexp}\left(-\frac{\text{Q}}{\text{RT}}\right)$$ (3)

Where, D is the bulk density, C is the characteristic constant of powder, K is the reaction rate constant, T is the sintering time, Q is the sintering activation energy, R is the gas constant, T is the absolute temperature, and A is the constant (Demirkiran et al., 2008; Yürüyen and Toplan, 2009).

**FIGURE 5:**
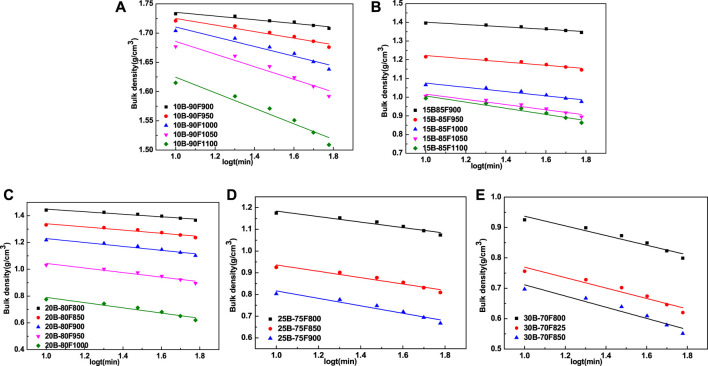
Bulk density (D) vs. log t graph for samples with different borax additions.

**FIGURE 6:**
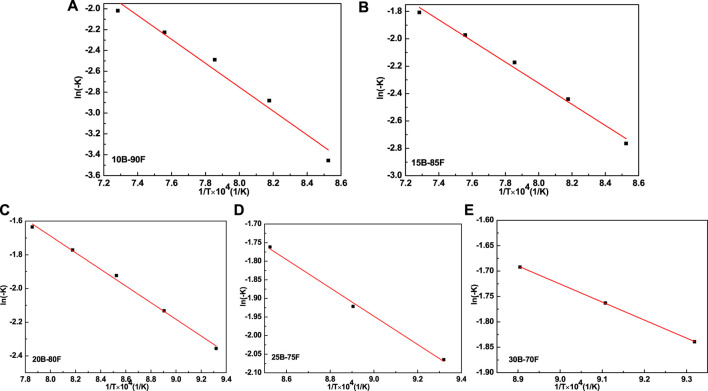
Ln (−k) vs. 1/T graph for samples with different borax additions.

Figure 5 is the curve of the logarithm of the bulk density and sintering time of porous glass-ceramics prepared with different borax additions. The bulk density of porous glass-ceramics is linearly correlated with the logarithm of sintering time, which conforms to the dynamic empirical formula of ceramic sintering (2). The relationship between ln (−k) and 1/T was plotted using the reaction rate constant K and sintering temperature T, as shown in Figure 6. According to Arrhenius formula, the sintering activation energy of porous glass-ceramics prepared by different borax additives was calculated, as shown in Table 3. As can be seen from the correlation factor in Table 3, the correlation factor is close to 1, indicating that ln (−k) has a good correlation with 1/T, and Arrhenius formula can be used to calculate the sintering activation energy. The sintering activation energy of porous glass-ceramics decreases with the increase of borax content, from 95.30 kJ/mol to 29.49 kJ/mol. Obviously, more borax is added, lower sintering activation energy is. On the one hand, the addition of borax provides more liquid phase to facilitate liquid phase sintering. On the other hand, the addition of borax provides B-O bond to change the network structure of non-crystalline vitreous of coal fly ash, to reduce its melting temperature, and promote liquid phase sintering.

**TABLE 3:** Activation energy of sintering samples with different borax additions.

Samples	Borax content (%)	Correlation factor (r)	−Q/R	Activation energy Q (kJ/mol)
10B-10F	10	0.98	11,462.50	95.30
15B-85F	15	0.99	7,715.40	64.15
20B-80F	20	0.99	4,944.10	41.11
25B-75F	25	0.99	3,809.70	31.67
30B-70F	30	0.99	3,547.00	29.49

**FIGURE 7:**
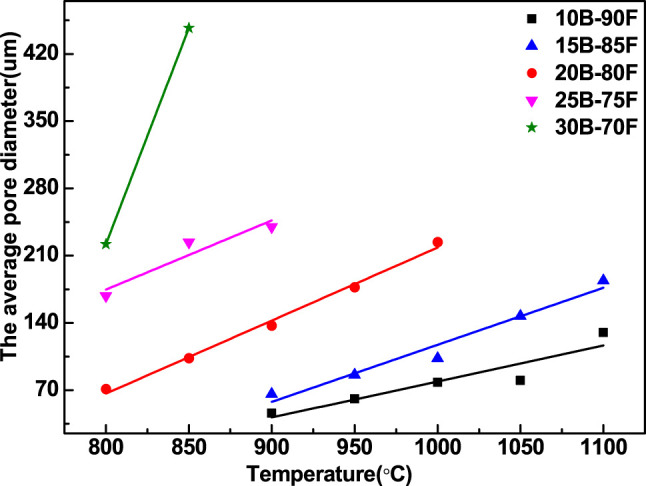
Relationship between average pore diameter of porous galss-ceramics with different borax additions and temperature.

Figure 7 shows the relationship between the average pore diameter and temperature of porous glass-ceramics prepared with different borax additions. With the increase of temperature and borax, the average pore size of porous glass-ceramics increased gradually. It can be seen from the figure that the average aperture has a good linear correlation with the temperature. With the increase of borax addition, the slope of the related line gradually increases. The more borax is added, the more B-O bonds are provided, the more liquid phase is provided, the easier sintering is, and the greater change of average pore diameter is. In other words, with the increase of borax addition, the mass transfer in liquid phase is more obvious, and the process of forming large pores is more affected by temperature. The sintering process of porous glass-ceramics by overfiring can be regarded as the fourth stage after the third stage of glass-ceramics, which is caused by mass transfer in liquid phase and the formation of large pores.

## Conclusion

In this experiment, the porous glass-ceramics was directly overfired from coal fly ash and borax at low temperature without pore-forming agent. It provides a new possibility for preparing porous glass-ceramics with low energy consumption and high utilization of coal fly ash. The addition of borax has great influence on the phase, morphology and properties of porous glass-ceramics. B-O bond in borax can destroy the structure of quartz and amorphous vitreous body in coal fly ash, reduce its melting temperature and increase the high-temperature liquid phase, thus increasing the content of anorthite. With the increase of borax content from 10% to 15%, the anorthite content in porous glass-ceramics also increases gradually. When the amount of borax increases to 15% and the sintering temperature is 1,100°C (15B-85F1100), the content of anorthite reaches the maximum. Then, with the increase of borax addition, the content of anorthite further decreased. When borax addition was 30% and sintering temperature was 850°C (30B-70F850), the content of anorthite decreased to zero. It shows that although borax can destroy the structure of quartz and amorphous vitreous in coal fly ash to precipitate anorthite, the role of sintering temperature in the preparation of porous glass-ceramics cannot be ignored. The larger the borax addition is, the larger average pore size and porosity of porous glass-ceramics are, the smaller the bulk density and the flexural strength are. The properties of porous glass ceramic can be adjusted by adjusting the sintering temperature or the amount of borax.

The dynamic process of direct overfiring for the preparation of porous glass-ceramics extends to the fourth stage after the three-stage theory of liquid phase sintering. That is, with the increase of temperature, the small pores remaining in the compact stage are merged into large pores due to the mass transfer effect of liquid phase. With the increase of borax, the sintering activation energy of porous glass ceramics decreased obviously. On the one hand, the addition of borax provides more liquid phase to facilitate liquid phase sintering. On the other hand, the addition of borax provides B-O bond to change the network structure of non-crystalline vitreous of fly ash, to reduce its melting temperature and promote liquid phase sintering.

## Data Availability

The original contributions presented in the study are included in the article/Supplementary Material, further inquiries can be directed to the corresponding author.
